# Which Food Security Determinants Predict Adequate Vegetable Consumption among Rural Western Australian Children?

**DOI:** 10.3390/ijerph14010040

**Published:** 2017-01-03

**Authors:** Stephanie L. Godrich, Johnny Lo, Christina R. Davies, Jill Darby, Amanda Devine

**Affiliations:** 1 School of Medical and Health Sciences, Edith Cowan University, 270 Joondalup Drive, Joondalup 6027, Australia; j.darby@ecu.edu.au (J.D.); a.devine@ecu.edu.au (A.D.); 2 School of Science, Edith Cowan University, 270 Joondalup Drive, Joondalup 6027, Australia; j.lo@ecu.edu.au; 3 School of Population Health, The University of Western Australia, 35 Stirling Highway, Crawley 6009, Australia; christina.davies@uwa.edu.au; 4 Public Health Advocacy Institute of Western Australia, Faculty of Health Sciences, Curtin University, GPO Box U1987, Perth 6845, Australia

**Keywords:** food security, vegetables, regional and remote Australia, child

## Abstract

Improving the suboptimal vegetable consumption among the majority of Australian children is imperative in reducing chronic disease risk. The objective of this research was to determine whether there was a relationship between food security determinants (FSD) (i.e., food availability, access, and utilisation dimensions) and adequate vegetable consumption among children living in regional and remote Western Australia (WA). Caregiver-child dyads (*n* = 256) living in non-metropolitan/rural WA completed cross-sectional surveys that included questions on FSD, demographics and usual vegetable intake. A total of 187 dyads were included in analyses, which included descriptive and logistic regression analyses via IBM SPSS (version 23). A total of 13.4% of children in this sample had adequate vegetable intake. FSD that met inclusion criteria (*p* ≤ 0.20) for multivariable regression analyses included price; promotion; quality; location of food outlets; variety of vegetable types; financial resources; and transport to outlets. After adjustment for potential demographic confounders, the FSD that predicted adequate vegetable consumption were, variety of vegetable types consumed (*p* = 0.007), promotion (*p* = 0.017), location of food outlets (*p* = 0.027), and price (*p* = 0.043). Food retail outlets should ensure that adequate varieties of vegetable types (i.e., fresh, frozen, tinned) are available, vegetable messages should be promoted through food retail outlets and in community settings, towns should include a range of vegetable purchasing options, increase their reliance on a local food supply and increase transport options to enable affordable vegetable purchasing.

## 1. Introduction

The Food and Agriculture Organisation states that food security incorporates the key dimensions of food availability, food access, food utilisation, and stability of these dimensions [1]. Each dimension includes a range of food security determinants (FSD). At the food availability level, key FSD include availability in outlets, price, promotion, quality; location of outlets; and variety [2,3]. Food access determinants include social support, household financial resources; transportation to outlets; distance to outlets; and mobility [2,3]. Food utilisation determinants include nutrition knowledge and skills; food preferences; household food storage facilities; cooking and food preparation facilities; and time to procure and prepare food [2,3,4].

Locations where availability and access to healthy food is difficult or absent, have been referred to as “food deserts” [5,6]. The limited food resources in such locations are often unaffordable and of poor quality [7]. These issues can negatively impact health [6] as a result of poorer dietary intake from important food groups such as vegetables [8].

Adequate vegetable consumption among children aged 9–11 and females aged 12–13 years is deemed by the 2013 Australian Dietary Guidelines (ADG) to be five or more serves, whereby a serve is equivalent to one cup of salad or half a cup of cooked vegetables. For males aged 12–13 years, the requirement increases to 5.5 serves per day [9]. Currently in Australia, the majority (almost 97%) of children consume inadequate amounts of vegetables [10]. Children living in Western Australia (WA) consume slightly higher amounts, however, the majority (almost 92%) do not achieve the recommended vegetable intake [11]. While evidence demonstrates most WA children are consuming inadequate amounts of vegetables, the way in which FSD impact children’s vegetable consumption remains largely unknown.

The majority of studies investigating the association between children’s vegetable consumption and food security have been limited to examining food security status [12,13,14,15,16,17] rather than FSD. Current evidence relating to FSD suggests the consumption of adequate vegetables is determined by cost, social support, location of food outlets, and transport to outlets. The cost of healthy food options has been suggested to be beyond the budget of many disadvantaged families [18,19,20]. Further, social support significantly increased fruit and vegetable intake among adolescents [21]. Children were also more likely to have infrequent vegetable consumption if they attend schools in locations with low supermarket density [22]. Residents living in neighbourhoods lacking in transport may have further difficulty accessing healthy food [23].

This study answers the call of previous research to examine how food security impacts dietary outcomes [3,24]. The aim of the current study was to determine whether FSD were associated with adequate vegetable consumption among regional and remote WA children.

## 2. Materials and Methods

### 2.1. Sampling and Recruitment

This study was conducted in non-metropolitan, rural areas of WA. Reference in this paper to “regional WA” and “remote WA” schools include the locations defined by the Australian Statistical Geography Standard [25]. That is, locations outside of WA’s metropolitan area encompassing “inner regional” and “outer regional” (herein referred to as “regional WA”), “remote” and “very remote” (herein referred to as “remote WA”) [25]. In Australia, the remoteness of locations is defined by an area’s access to services [25]. Schools who were eligible to participate in the Foodbank WA Food Sensations^®^ program were invited. Children aged 9–13 years and their caregivers were selected to facilitate comparisons between children’s vegetable intake and the ADG recommendations for fruit and vegetables (F & V) [26]. The WA Department of Education (DOE) annual student census was used to inform a sample size calculation for the research question relating to this manuscript, and determined the sample required (*n* = 160 children and 160 of their caregivers based on an effect size of 0.15 (small), *α* = 0.05, 80% power) [27]. School authority websites [28,29,30] were used to compile a Master Schools Database which listed schools by WA region [31] (e.g., Pilbara), remoteness [32], and Socio-economic Index for Areas (SEIFA) Index of Relative Socio-economic Disadvantage (IRSD) score [33].

School principals were initially engaged via an introductory telephone call to explain the study, followed by an email containing a principal Invitation Letter (IL) and Consent Form (CF), in addition to a DOE approval letter. Almost three-quarters (72%, *n* = 23) of the school principals invited to participate consented for their school to participate. Principals nominated classes of students aged between 9 and 13 years (*n* = 71 teachers of 76 classes) on their principal CF. Almost all (97%, *n* = 69 teachers, 74 classes) teachers invited via a teacher IL returned the signed teacher CF to the study centre. Where possible (*n* = 51 classes), a teacher and class briefing session was delivered, explaining the study to classes, disseminating the caregiver and child IL/CF envelopes, and providing survey packs to teachers. Teachers of the 23 classes that did not participate in a briefing session were mailed study packs for dissemination. A total of 1814 caregivers and their children were invited to participate in the study, with 347 caregivers and 340 children providing written informed consent. A child-caregiver dyad was the chosen method to facilitate comparisons between data in the wider study, and given matched caregiver and child surveys were a requirement for inclusion, a total of 256 dyads were included in the sample. Due to missing data for some study variables, a total of 187 dyads have been included in analyses.

### 2.2. Instruments

#### 2.2.1. Socio-Demographic Questions

The research team developed cross-sectional, self-administered, pictorial, paper-based child and caregiver surveys. Socio-demographic questions included caregiver and child age, gender, caregiver educational attainment, etc., and are summarised in Table 1. For example, the caregiver survey enquired about caregiver educational attainment, including response options of “primary school”; “secondary school”; “apprenticeship or diploma”; “university degree” and “postgraduate university degree”.

#### 2.2.2. Independent Variable Questions

FSD were the independent variables in this study. FSD across community-level food availability, household-level, and individual-level food access and utilisation dimensions were measured by questions underpinned by the Determinants of Food Security [2] model. These were based on previous research [34] and investigator-initiated questions (Table 2). For example, transportation modes to access F & V included “car”, “bus”, “walk”, “bicycle”, “no transport”, or “other” response options. Some FSD variables were measured using a five-point Likert scale of “strongly agree”, “agree”, “unsure”, “disagree”, or “strongly disagree”. For example, caregiver knowledge and skills were measured via level of agreement with the statement: “I don’t know how to use vegetables in meals”. Number of vegetable types consumed in the previous month was measured through the question “Please tick which type of vegetables your child ate in the previous month”. Options included “fresh”, “frozen”, “tinned”, “dried”, and “juice”. All vegetable types included the response options “yes” or “no”.

#### 2.2.3. Dependent Variable Question

“Adequate vegetable consumption” was the dependent variable in this study. The question measuring usual vegetable serves consumed by children was based on the WA Child and Adolescent Physical Activity and Nutrition Survey [35] (used with permission). Children’s usual daily vegetable serves, as reported by their caregiver, were measured using the question “How many serves of vegetables does your child usually eat each day?” Prompts were provided to outline what constitutes a serve of vegetables (i.e., one cup of salad vegetables). Response options included: “My child doesn’t eat vegetables”; “one serve or less each day”; “2 serves each day”; “3 serves each day”; “4 serves each day”; “5 serves each day”; “6 or more serves each day”; “don’t know”. Responses were compared with the ADG recommendation [26] to ascertain whether intake was “adequate” (≥5 serves of vegetables each day) or “inadequate” (<5 serves of vegetables each day). As these survey tools were developed prior to the release of the 2013 ADG, information relating to half serves was not collected. Therefore, male children aged 12 and 13 years were deemed to have consumed adequate vegetable serves if they consumed ≥5 serves instead of the recommended ≥5.5 serves.

### 2.3. Data Collection

Data collection commenced in March 2013 and concluded in December 2015. This included a pilot phase where face validity and reliability testing were conducted and confirmed in one school with 26 dyads. The child survey was completed in class with their class teacher, with completed surveys sealed by students for privacy. Caregiver surveys were completed at home, with a sealable envelope provided for privacy. Schools returned all CF and completed surveys to the study centre in a pre-paid postal envelope. Teachers and caregivers provided written feedback regarding information provided about the study, question wording, convenience of the study processes, and suggestions for improvement.

### 2.4. Data Analysis

A unique Identification (ID) number was allocated to each caregiver-child dyad, with child and caregiver surveys entered into separate, password-protected Microsoft Excel datasets. Datasets were imported into IBM SPSS (IBM, Armonk, NY, USA) for analyses. Any cases with missing data were excluded in analyses; only complete cases remained (*n* = 187). Caregiver-reported FSD and vegetable intake of their children was included in this paper.

#### 2.4.1. Variable Recoding

Due to low cell counts, a number of socio-demographic variables required recoding, including caregiver educational attainment, caregiver employment status, SEIFA IRSD decile, and number of household residents. For example, caregiver educational attainment was recoded to “primary school or secondary school”, “diploma or apprenticeship” or “undergraduate/postgraduate university degree” (Table 1). Independent variables that required recoding included variables with five-point response options (i.e., “Strongly agree”, “Agree”, “Unsure”, “Disagree”, or “Strongly disagree”) (Table 2).

#### 2.4.2. Simple Regression Analyses

The relationship between each of the independent variables and the outcome variable were assessed via simple logistic regression analyses. All independent variables were entered as categorical variables except for number of residents in the household, distance to food outlets, and time required to travel to food outlets, which were entered as continuous variables. Potential confounding variables including caregiver age and child age were entered as continuous variables. Inclusion criterion for entry into multivariable analyses was a conservative significance level of *p* ≤ 0.20. The use of a conservative inclusion criteria is a validated approach [36] and was used to elucidate important variables for inclusion in the multivariable analyses, as has been used in other studies [37].

#### 2.4.3. Multivariable Regression Analyses

Significant FSD variables identified in the simple logistic regression analyses were entered into two multivariable models (Table 3), the latter of which was controlled for socio-demographic factors demonstrated in the literature to be associated with adequate vegetable consumption, such as caregiver educational attainment [38], SEIFA [39,40], and child gender [39]. Child age [22,39], caregiver age [41], caregiver gender [41], and remoteness [42,43] were also included as potential confounders. The level of significance was set at *p* ≤ 0.05.

#### 2.4.4. Ethical Approval

All subjects gave their informed consent for inclusion before they participated in the study. The study was conducted in accordance with the Declaration of Helsinki, and was approved by the Edith Cowan University Human Research Ethics Committee (project identification code 8635).

## 3. Results

### 3.1. Demographics of Sample

The majority of caregiver respondents were female (85.0%) with an age range of 26–63 years and a mean age of 40.6 years (SD = 6.0). Overall, 59.4% of the respondents were from regional WA, while 40.6% were from remote WA. The highest level of education attained by almost half of the respondents (42.2%) was completion of primary (junior, previously in WA year 1 to 7) or secondary (senior, year 8–12) school. Over two-thirds of the child sample was females, while the average age of children was 10.9 years (the equivalent of the final year of junior/primary school in WA). A total of 64.7% of families lived in locations deemed as having a high level of socio-economic disadvantage.

### 3.2. Food Security Determinants across Food Availability, Access, and Utilisation Dimensions

The results of this study highlighted the inequalities associated with living in regional and remote WA. Over half (50.3%) of the respondents indicated they would eat healthier food if their food outlets stocked healthier options, while 79.1% believed food in their community cost more than other communities. One-third indicated food quality was suboptimal. The importance of informal social support networks was highlighted by 78.1% of respondents indicating they would turn to a family member or friend if they were having difficulty feeding their family; however, almost one in five (17.1%) would not tell anyone. The majority of caregivers reported knowing how to incorporate vegetables into meals, while 11.8% agreed/were unsure whether their child disliked the taste of vegetables (Table 2).

### 3.3. The Association between Food Security Determinants and Vegetable Consumption

A total of 13.4% of children in this study sample had adequate vegetable intake. Variables that met the inclusion criteria (*p* ≤ 0.20) for multivariable regression analyses included caregiver agreement that healthy food cost more in their community (FSD of price); caregiver recall of a promotional health message or slogan relating to vegetables (Promotion); caregiver agreement that they would eat more vegetables if they did not spoil so often (Quality); agreement that there were enough food outlets in their community (Location of food outlets); number of vegetable types consumed by the child (Variety); family receipt of government income support (Financial resources); and number of transport modes used by the family to purchase vegetables (Transport to food outlets) (Table 2). After inclusion in multivariable analyses, significant determinants (significant at *p* ≤ 0.05) that predicted adequate vegetable intake in the adjusted model included number of vegetable types consumed by the child (Variety) (*p* = 0.007); caregiver recall of a promotional health message or slogan relating to vegetables (Promotion) (*p* = 0.017); caregiver reported agreement that there are enough food stores in their community (Location of food outlets) (*p* = 0.027); and caregiver reported agreement that the cost of healthy eating is higher in their community than other places (*p* = 0.043) (Price). Children who consumed four to five different types/forms of vegetables (i.e., fresh, frozen, tinned, dried, juice) were approximately ten times more likely to consume adequate amounts of vegetables (≥5 serves) [26] compared to those that consumed one or two types. Children whose caregivers recalled a promotional vegetable message or slogan were approximately five times more likely to consume adequate amounts of vegetables for good health, compared to children whose caregivers did not recall a message. Children of caregivers that agreed there were enough food outlets in their town were approximately ten times more likely to eat enough vegetables, while caregivers who believed the cost of healthy eating was not higher in their town, compared to other towns, were approximately three times more likely to have children that consumed adequate vegetables. Number of transport modes used by the family to purchase vegetables (Transport to food outlets) was weakly associated with adequate vegetable consumption (*p* = 0.063). Children whose family used three transport modes to purchase vegetables were more likely to eat enough vegetables in comparison to children whose family used only one transport mode (Table 3). Child age was the only significant confounding variable in this model (*p* = 0.022), in that children’s consumption reduced with age.

## 4. Discussion

The aim of this research was to determine whether FSD were associated with adequate vegetable consumption among regional and remote WA children. The significant determinants that predicted adequate vegetable consumption at the multivariable level were within the food availability dimension and included children’s consumption of four to five vegetable types/forms; caregiver recall of a promotional health message or slogan relating to vegetables; presence of sufficient food outlets in their town; and similar vegetable prices to other towns/communities. The number of transport options used by families to purchase vegetables was weakly associated with adequate vegetable consumption among children.

The importance of controlling for potential confounding variables was highlighted in this study. The number of transport modes was not a significant predictor in the unadjusted model, yet after adjustment, was significant at *p* ≤ 0.10. A number of FSD increased in significance from *p* ≤ 0.10 to *p* ≤ 0.05 between the unadjusted and adjusted models (food price, location of food outlets). FSD that remained significant between unadjusted and adjusted models included promotion and variety of vegetable types, indicating these factors are key drivers of vegetable consumption regardless of socio-demographic factors. In contrast, after adjustment, financial resources no longer significantly predicted children’s vegetable intake.

Inclusion of a range of vegetable types in the diet, such as fresh, frozen, and tinned, are all recommended for good health by the ADG [26]. Previous research indicated availability, cost, and quality of fresh vegetables is a critical issue for regional and remote areas [4,44,45]. Therefore ensuring a range of alternative vegetable types including frozen and tinned offers more opportunities for children to consume adequate quantities of vegetables at a more affordable cost with fewer quality issues than fresh vegetables [46]. Further, many of these types are convenient and may be more readily available when their fresh counterparts are out of season [4,47].

Our finding that 13.4% of children within this study sample met the ADG for vegetables was slightly higher than other Western Australian data, which found that 8.8% of children met vegetable guidelines [11].

Our finding that caregiver recall of a vegetable promotional message increased the likelihood of adequate vegetable consumption was consistent with previous evidence. The “Go for 2&5^®^” campaign national evaluation measured recall of campaign messages [48]. Almost half of the parents surveyed indicated the campaign prompted them to take action to improve their family’s vegetable consumption, such as increasing the vegetable quantities their family consumed, adding an extra serve of vegetables, or employing the use of vegetable based recipes. Actions to increase their family’s vegetable consumption were significantly higher in the second follow up survey of the national evaluation, compared to the baseline survey [48]. Among WA adults, vegetable consumption increased by 0.6 serves per person during the life of the “Go for 2&5^®^” campaign [49,50]. With regards to communication channels to promote health messages, recognition of vegetable promotional messages in the New South Wales “Eat It To Beat It” campaign were highest for school or other newsletter articles (44%), television or community announcement (42%), recipe cards (24%), and vegetable recipe demonstrations (14%) [51]. Exposure to each additional promotional strategy resulted in a significant increase in vegetable serves [51].

Our finding that sufficient food outlets predicted adequate vegetable consumption concurred with previous literature. Adequate food outlets located in towns are key drivers of food purchasing and consumption decisions [2], with poor density of food outlets shown previously to be associated with inadequate vegetable consumption among adolescents [22].

Strengths of this study include, to our knowledge, the first investigation in Australia to measure the relationship between a wide range of FSD and adequate vegetable consumption. This study sample also comprised participants living in all WA Remoteness Areas, and the majority of WA regions and levels of disadvantage. Additionally, the investigation of vegetable consumption through a FSD lens increases understanding about the relationship between FSD and vegetable consumption. This is particularly useful for advocacy, practice, and research efforts to improve “food deserts”, such as in some regional and remote areas. However, there were a number of limitations associated with this study. The low participation rate was suggested to be, in part, a result of the consent processes required for study approval. Active written consent was required from school principals, each class teacher, each caregiver, and each child. Further, children/caregivers were excluded from the study if their child/caregiver counterpart did not participate. This negatively impacted the sample size included in analyses.

This research highlighted the range of FSD that can affect a child’s likelihood of consuming adequate amounts of vegetables for good health. Our study findings suggest resulting points of intervention should occur primarily within the food and nutrition system, such as in food outlets (i.e., supermarkets) or direct retail options such as farmers’ markets [2]. Recommendations relating to significant determinants identified in this study (significant at *p* ≤ 0.05) include (i) increasing the range and promotion of vegetable types (i.e., fresh, frozen, tinned, dried, juice) available to and within local food retail outlets across regional and remote WA. This could increase the likelihood of children living in these locations consuming adequate vegetables; (ii) long-term, consistent promotion of specific vegetable messages utilising a wide range of promotional strategies; (iii) consideration of town planning to enable multiple food retail options (i.e., supermarkets, farmers’ markets, produce stalls) [4] for regional and remote families to source vegetables from, positioned in accessible locations within the town. In addition, consideration of community gardens or edible landscapes could increase opportunities to source and consume adequate vegetables; (iv) increased reliance on a local food supply to reduce the cost of vegetables in regional and remote locations, or core food freight subsidies. Table 4 provides detailed implementation strategies for each of the significant determinants of adequate vegetable consumption. Due to weak associations, cautious recommendations relating to the issue of “transport” could be considered and could include increasing the number of transport options available in towns to increase the potential for families to access food [2,23,52]. For example, recommendations could be made for increasing availability or efficiency of public transport, or changes to the built environment to facilitate more active transport. As this paper only reports on investigations into vegetable consumption, further research could “complete the picture” by ascertaining which FSD impact children’s fruit consumption. Although approximately two-thirds of WA children are consuming adequate amounts of fruit [11], further investigation of the factors that predict fruit consumption could improve intake among the current one-third of children consuming suboptimal amounts of fruit [11].

## 5. Conclusions

This study makes a significant contribution to literature through its investigation into the key FSD impacting regional and remote WA children’s vegetable consumption. Action taken to implement the recommendations and associated strategies suggested by this research, relating to increasing availability of a range of vegetable types, promotion, increasing range of food outlets, reduced price, and increasing transport options, may assist in increasing the largely inadequate vegetable consumption among children living in regional and remote WA.

## Figures and Tables

**Table 1:** Simple logistic regression models for confounding variables/socio-demographic factors and adequate vegetable consumption, among regional and remote Western Australian children (*n* = 187).

Confounding Variables—Socio-Demographic Factors		Adequate Vegetable Consumption		
		Total *n* (%)	OR **^#^**	*p*-Value
			(95% CI **^^^**)	
Caregiver age (years)	26–63 years	40.6 ^†^	1.02 (0.95, 1.10)	0.437
Caregiver gender	Male	28 (15.0)	1.51 (0.51, 4.42)	0.452
	Female	159 (85.0)	1.00 (ref)	
Caregiver highest level of educational attainment	Overall			0.311
	Primary school/Secondary school	79 (42.2)	1.00 (ref)	
	Diploma/Apprenticeship	59 (31.6)	2.09 (0.74, 5.89)	0.159
	Undergraduate University degree/Post-graduate University degree	49 (26.2)	2.00 (0.67, 5.93)	0.208
Child age (years)	9–13	10.9 ^†^	0.77 (0.51, 1.17)	0.225
Child gender	Male	64 (34.2)	1.09 (0.45, 2.63)	0.841
	Female	123 (65.8)	1.00 (ref)	
SEIFA IRSD range **^1^**	Overall			0.450
	High disadvantage	121 (64.7)	1.00 (ref)	
	Medium disadvantage	49 (26.2)	0.50 (0.16, 1.58)	0.245
	Low disadvantage	17 (9.1)	1.22 (0.32, 4.70)	0.766
Geographical location **^2^**	Regional	111 (59.4)	1.03 (0.43, 2.43)	0.944
	Remote	76 (40.6)	1.00 (ref)	

OR **^#^** = Odds Ratio; CI **^^^** = Confidence Interval; **^†^** Mean; 1.00 (ref) = reference category; **^1^** SEIFA (Socio-economic Index for Areas) Low score (High disadvantage) includes IRSD (Index of Relative Socio-economic Disadvantage) scores of 1–3; Medium score (Medium disadvantage) includes IRSD scores of 4–6; High score (Low disadvantage) includes IRSD scores of 7–10; **^2^** Geographical location “regional” includes the Australian Statistical Geography Standard Remoteness Areas (ASGS RA) of “inner regional” and “outer regional” [25]; geographical location of “remote” includes the ASGS RA of “remote” and “very remote” [25].

**Table 2:** Simple logistic regression models for food security determinants and adequate vegetable consumption, among regional and remote Western Australian children (*n* = 187).

Food Security Dimension	Food Security Determinant	Description	Response	Adequate Vegetable Consumption		
				Total *n* (%)	OR **^#^** (95% CI **^^^**)	*p*-Value
**Food Availability**	**Availability in Outlets**	Caregiver reported agreement that they would eat healthier food if more healthy options were available in their community’s stores ^**1**^	Disagree	93 (49.7)	1.33 (0.57, 3.12)	0.502
			Agree/Unsure	94 (50.3)	1.00 (ref)	
	**Price**	Caregiver reported agreement that the cost of healthy eating is higher in their community than other places **^1^**	Disagree	39 (20.9)	1.98 (0.78, 5.02)	0.146 **^+^**
			Agree/Unsure	148 (79.1)	1.00 (ref)	
	**Promotion**	Caregiver recall of a promotional health slogan or message relating to vegetables	No	66 (35.3)	1.00 (ref)	
			Yes	121 (64.7)	3.25 (1.06, 9.92)	0.038 **^+^**
	**Quality**	Caregiver reported agreement that they would eat more vegetables if they did not spoil so often **^1^**	Disagree	124 (66.3)	3.00 (0.98, 9.18)	0.053 **^+^**
			Agree/Unsure	63 (33.7)	1.00 (ref)	
	**Location of Food Outlets**	Caregiver reported agreement that there are enough food stores in their community **^1^**	Unsure/Disagree	43 (23.0)	1.00 (ref)	
			Agree	144 (77.0)	3.89 (0.88, 17.25)	0.073 **^+^**
	**Variety**	Number of vegetable types consumed by child in past month **^2^**	Overall			0.105 **^+^**
			1–2	86 (46.0)	1.00 (ref)	
			3	75 (40.1)	0.90 (0.33, 2.43)	0.847
			4–5	26 (13.9)	2.80 (0.94, 8.31)	0.064
**Food Access**	**Social Support**	Who caregiver would tell if they were finding it difficult to feed their family	Overall			0.649
			No-one	32 (17.1)	1.00 (ref)	
			Informal Support (Family/friend)	146 (78.1)	0.75 (0.25, 2.22)	0.616
			Formal Support (School/Agency)/both Informal and Formal social support	9 (4.8)	1.54 (0.24, 9.70)	0.644
	**Financial Resources**	Family receipt of government income support	No	141 (75.4)	2.65 (0.75, 9.30)	0.128 **^+^**
			Yes	46 (24.6)	1.00 (ref)	
		Caregiver employment status	Overall			0.743
			Unemployed/Volunteer	31 (16.6)	1.00 (ref)	
			Part time	77 (41.2)	1.39 (0.35, 5.44)	0.634
			Full time	79 (42.2)	1.67 (0.43, 6.38)	0.452
		Number of household residents	2–14	4.6 (100)	0.87 (0.60, 1.27)	0.877
	**Transport to Food Outlets**	Number of transport modes used to purchase vegetables **^3^**	Overall			0.129 **^+^**
			1	129 (69.0)	1.00 (ref)	
			2	40 (21.4)	0.53 (0.14, 1.92)	0.338
			3	18 (9.6)	2.53 (0.80, 8.00)	0.113
	**Distance to Food Outlets**	Distance to food outlet to purchase vegetables (km)	0–200 km	11.0 **^†^**	0.99 (0.96, 1.01)	0.495
**Food Utilisation**	**Nutrition Knowledge and Cooking Skills**	Caregiver reported agreement that they do not know how to use vegetables in meals	Disagree	184 (98.4)	N/A	0.999
			Agree/Unsure	3 (1.6)	1.00 (ref)	
	**Food Preferences**	Caregiver reported agreement that their children don’t like the taste of vegetables	Disagree	165 (88.2)	3.57 (0.45, 27.82)	0.224
			Agree/Unsure	22 (11.8)	1.00 (ref)	
	**Storage Facilities**	Household storage facilities available **^4^**	Less than three food storage options	4 (2.1)	1.00 (ref)	
			Three food storage options	183 (97.9)	0.45 (0.04, 4.53)	0.500
	**Food Preparation and Cooking Facilities**	Household food preparation and cooking facilities used **^5^**	Gas/electrical appliances only	151 (80.7)	1.00 (ref)	
			Fire and gas/electrical appliances	36 (19.3)	1.05 (0.36, 3.03)	0.919
	**Time**	Time required to travel to food outlets (minutes)	0–120 min	7.89 **^†^**	0.99 (0.96, 1.03)	0.919

**^#^** OR = Odds Ratio; CI **^^^** = Confidence Interval; 1.00 (ref) = reference category; **^+^** Significant at *p* ≤ 0.20. Included in multivariable model; **^†^** Mean; N/A = Estimates unavailable due to low counts of SA/A/Unsure; **^1^** Questions sourced from Hendrickson, D., Smith, C., Eikenberry, N. (2006) [34]; **^2^** Vegetable types included “Fresh”, “Frozen”, “Tinned”, “Dried”, “Juice”; **^3^** Number of transport modes includes the sum of “Car”, “Bus”, “Bicycle”, and “Walk” options. Note: no respondents reported using all four transport modes; **^4^** Household storage facilities includes the sum of “Refrigerator”, “Freezer”, “Cupboard/pantry” options (either all three options or less than three options); **^5^** Household food preparation and cooking facilities includes the sum of gas/electrical appliances: “Stove/cook top”, “Oven”, “Barbecue”, “Microwave”, and sum of gas/electrical appliances plus “Open fire”.

**Table 3:** Multivariable logistic regression models for food security determinants and adequate vegetable consumption, among regional and remote Western Australian children (*n* = 187).

Food Security Dimension	Food Security Determinant	Description	Response	1. Adequate Vegetable Consumption (Unadjusted Model)		2. Adequate Vegetable Consumption (Adjusted for Socio-Demographic Factors)	
				OR **^#^** (95% CI **^^^**)	*p*-Value	OR **^#^** (95% CI **^^^**)	*p*-Value
**Food Availability**	**Price**	Caregiver reported agreement that the cost of healthy eating is higher in their community than other places **^1^**	Disagree	2.56 (0.85, 7.74)	0.095 *	3.79 (1.04, 13.87)	0.043 **
			Agree/Unsure	1.00 (ref)		1.00 (ref)	
	**Promotion**	Caregiver recall of a promotional health slogan or message relating to vegetables	No	1.00 (ref)		1.00 (ref)	
			Yes	3.83 (1.14, 12.84)	0.029 **	5.62 (1.36, 23.20)	0.017 **
	**Quality**	Caregiver reported agreement that they would eat more vegetables if they did not spoil so often **^1^**	Disagree	2.40 (0.69, 8.29)	0.164	1.99 (0.49, 8.08)	0.331
			Agree/Unsure	1.00 (ref)		1.00 (ref)	
	**Location of Food Outlets**	Caregiver reported agreement that there are enough food stores in their community **^1^**	Unsure/Disagree	1.00 (ref)		1.00 (ref)	
			Agree	5.08 (0.98, 26.31)	0.052 *	10.29 (1.30, 81.43)	0.027 **
	**Variety**	Number of vegetable types consumed by child in past month **^2^**	Overall		0.017 **		0.007 **
			1–2	1.00 (ref)		1.00 (ref)	
			3	0.89 (0.30, 2.57)	0.829	1.10 (0.35, 3.44)	0.868
			4–5	5.72 (1.51, 21.62)	0.010	10.30 (2.22, 47.69)	0.003
**Food Access**	**Financial Resources**	Family receipt of government income support	No	3.72 (0.87, 15.80)	0.074 *	2.22 (0.44, 11.23)	0.332
			Yes			1.00 (ref)	
	**Transport to Food Outlets**	Number of transport modes used to purchase vegetables **^3^**	Overall		0.132		0.063 *
			1	1.00 (ref)		1.00 (ref)	
			2	0.42 (0.10, 1.76)	0.239	0.37 (0.07, 1.81)	0.223
			3	2.58 (0.67, 9.97)	0.168	3.95 (0.79, 19.63)	0.093

OR **^#^** = Odds Ratio; CI **^^^** = Confidence Interval; 1.00 (ref) = reference category; ** Significant at *p* ≤ 0.05; * Significant at *p* ≤ 0.10; **^1^** Questions sourced from Hendrickson, D., Smith, C., Eikenberry, N. (2006) [34]; **^2^** Vegetable types included “Fresh”, “Frozen”, “Tinned”, “Dried”, “Juice”; **^3^** Number of transport modes includes the sum of “Car”, “Bus”, “Bicycle”, and “Walk” options. Note: no respondents reported using all four transport modes; Nagelkerke R Square statistic was 0.363; The *p*-value of the Hosmer and Lemeshow Goodness of Fit Test was 0.982.

**Table 4:** Recommendations and implementation strategies to increase regional and remote Western Australian children’s vegetable consumption, based on key findings from this study and previous research.

Recommendation	Setting	Strategies
**(i) Increasing the range and promotion of vegetable types/forms (i.e., fresh, frozen, tinned, dried, juice) available to and within local food outlets**	Local food retail outlets (i.e., supermarket, farmers’ markets, online)	Training of food outlet owners/managers regarding selection, stocking, pricing, and maintenance a range of vegetable types [53] could be undertaken through management and/or a nutritionist working with the food outlet [44,54]. Purchasing and consuming less well-promoted types (i.e., tinned vegetables) could be promoted through positioning these types in easy-to-locate areas of the food outlet, online, etc. [2,54].
	Any settings where health practitioners work	Health practitioners should promote consumption of a range of vegetable types (“low sodium”/“no added salt” versions of tinned vegetables) with families and children, which is consistent with the ADG recommendations [26]. This may increase community requests or advocacy [2] for a range of vegetable types. Further, it may assist children to achieve the recommended vegetable quantities for good health [26].
**(ii) Long-term, consistent promotion of specific vegetable messages utilising various promotional strategies**	Local food retail outlets (i.e., supermarket, prepared food outlets, farmers’ markets, online)	Government-funded reinstatement of the “Go for 2&5^®”^ campaign in WA, or development of a similar vegetable promotional campaign with clear, consistent, action-based vegetable messages. The campaign should target parents and families, with promotional paraphernalia provided free of charge to food outlets, farmers’ markets, etc. Promotional strategies to disseminate vegetable messages could include Point-of-Purchase information such as shelf labels/talkers [54], provision of in-store/online recipe cards [55], in-store radio and regular “specials” [2], or locally created posters [4,56,57]. This should be coupled with interactive strategies [55,56] such as supermarket/market tours [54] and recipe demonstrations incorporating seasonal vegetables and promoting identified messages [57]. Promotional strategies for core foods (i.e., vegetables) should also be adopted for online shopping through supermarket websites.
	Schools, out-of-school care centres, community centres, other	Local strategies should support mass media campaigns and local media promoting vegetable consumption (i.e., through community announcements on radio and television) [2]. Food outlet vegetable promotion should be reinforced by promotion in settings-based interventions where parents are engaged (i.e., schools, out-of-school care centres, community centres) [24,57] and also be delivered with children (i.e., the “Crunch & Sip ^®^” program in schools) [58]. Credible health agencies endorsing interventions across settings is an effective strategy [56].
**(iii) Increase opportunities for families to acquire vegetables from multiple sources in their town**	Local food outlets (i.e., supermarket, prepared food outlets, farmers’ markets, online); community settings	Consideration of town planning to enable multiple food retail options (i.e., supermarkets, farmers’ markets, produce stalls) [4] for regional and remote families to source vegetables from, in accessible locations within the town [2,42,43]. Consideration of community gardens or edible landscapes [2].
**(iv) Increased reliance on a local food supply to reduce cost as a barrier to vegetable consumption**	Local food outlets (i.e., supermarket, prepared food outlets, farmers’ markets, online)	Increased reliance on a local food supply [4,52] to reduce the cost of vegetables, or core food freight subsidies [3,45,59].
